# Intra-articular corticosteroid injections in juvenile idiopathic arthritis

**DOI:** 10.1186/1546-0096-12-S1-P12

**Published:** 2014-09-17

**Authors:** Roberto Carrasco, Susannah Holt, Helen Foster, Lucy Wedderburn, Alice Cheing, Joyce Davidson, Yiannis Ioannou, Kimme Hyrich, Wendy Thomson, Eileen Baildam

**Affiliations:** 1 Arthritis Research UK Centre for Genetics and Genomics Centre for Musculoskeletal Research Institute of Inflammation and Repair Manchester , Manchester Medical School, Manchester, UK; 2 Palliative Care, Alder Hey Children's Foundation NHS Trust, Liverpool, UK; 3 Paediatric Rheumatology, Newcastle Royal Infirmary, Newcastle, UK; 4 Paediatric Rheumatology and Immunology, Institute of Child Health, London, UK; 5 Paediatric Rheumatology, Manchester Royal Children's Hospital, Manchester, UK; 6 Department of Paediatric Rheumatology, Edinburgh Children's Hospital, Edinburgh, UK; 7 Adolescent Rheumatology, University College Hospital, London, UK; 8 Department of Paediatric Rheumatology, Alder Hey Children's Foundation NHS Trust, Liverpool, UK

## Introduction

Intra-articular corticosteroid injections (IACI) are a standard treatment in juvenile idiopathic arthritis (JIA).

## Objectives

This study aims to assess the use and response to IACI in a large prospective cohort of children and young people (CYP) recruited at initiation of treatment for JIA.

## Methods

Participants were in the Childhood Arthritis Prospective Study (CAPS), an on-going prospective inception cohort study in 7 UK paediatric rheumatology centres. The aim of CAPS is to provide long-term outcome data on CYP with new-onset inflammatory arthritis receiving specialist care. CAPS recruits CYP <16 years with new inflammatory arthritis persisting for ≥ 2 weeks. Demographics, disease features, joint count, treatment details, Childhood Health Assessment Questionnaire (CHAQ), physician’s global assessment (PGA), parent’s general evaluation of well-being (PGE), ESR are collected at first presentation, 6 months, then yearly.

## Results

Of the 1477 CYP recruited to CAPS 759 have completed 3 years follow-up. 603 (79.5%) were treated with intra-articular cortico-steroid injections. 185 (24.4%) patients required IACI alone (with a single episode of injection as the only treatment in 100, (13 % of the total cohort) usually the knee in 80 %. The majority of injected patients went on to receive additional treatments, 393 (69.3%) commenced a disease-modifying anti-rheumatic drug or biologic agent. Of these, 93 patients received both DMARD/ biologic and IACI at the same time. No IACIs were received in 156 patients (20.55%) with 71 of these patients receiving oral or intra-venous steroids, 88 DMARDs and 27 biologic drugs.

Of the 185 patients treated only with IACI, 85 had more than one episode of injections. For this group the median time to first injection was 14 days (IQR 6.36) and time from first to second injection was 318 days ( IQR 162-525) illustrating a prolonged effect from the first injection.

390 of the 759 patients completing 3 years of follow-up had oligoarticular JIA of whom 332 (85%) received steroid injections, 163 (42%) treated exclusively with IACI 85 (25%) receiving only one episode of injection.Baseline predictors of the need for DMARD in addition to IACI were a higher total active and limited joint counts, ESR, physician’s global and the CHAQ score (p<0.0001), and pain scores (p<0.003).

## Conclusion

The data confirms that most children with JIA receive IACI usually in conjunction with other therapies. Approximately one quarter of patients required monotherapy with IACI alone. 13% of all patients and 25% of oligo-articular course patients were managed with a single injection alone. Higher measures of disease activity were significantly associated with the need for DMARD therapy in addition to IACI.

## Disclosure of interest

None declared.

